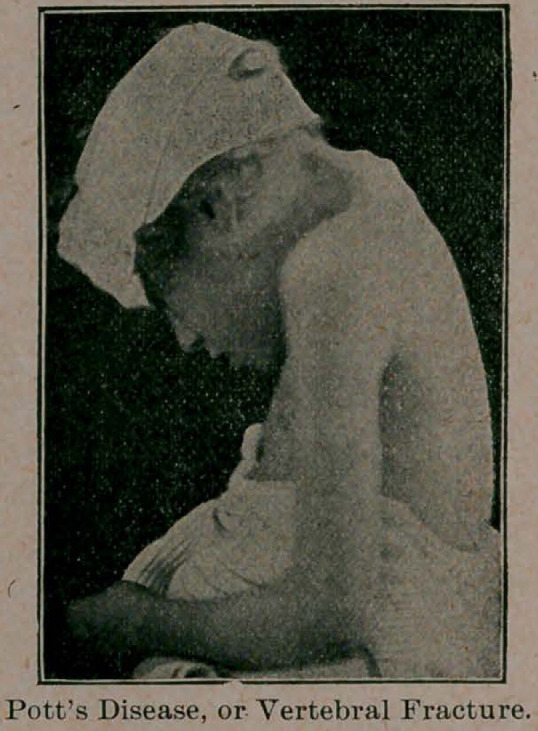# New York Academy of Medicine

**Published:** 1900-01

**Authors:** 


For Texas Medical Journal.

### New York Academy of Medicine.—Section on Orthopaedic Surgery.—Meeting of Nov. 17, 1899.

---

### FRACTURE ADDING TO THE DEFORMITY OF POTT'S DISEASE.

Dr. W. R. Townsend presented a boy, 15 years of age, who developed Pott's disease in the lower dorsal and upper lumbar region six years ago. Two years ago, having recovered with considerable posterior curvature, after treatment by the plaster of Paris jacket, he fell from an ice wagon, striking on his head. Plaster of Paris was re-applied. He presented a projection on each side at about the level of the twelfth dorsal vertebra. The spinous processes could be felt between the elevations which were very marked and might have been supposed to be calluses following fracture of the ribs near vertebral column.

Dr. R. H. Sayre said that in addition to the antero-posterior curve there was lateral displacement which might well have been the result of vertebral fracture.

Dr. S. Ketch* said that the bony projections were secondary formations, the result of traumatism and distinct from the spinal disease.

RICKETS IN A DWARF.

Dr. Townsend presented a girl, 6 years of age, 35 inches in height, the average height at that age being between 40 inches and 42 inches. There was enlargement of the epiphyses of the long bones with an enlarged head, prominent chest and protruding abdomen. She was a mouth-breather and failure to grow normally might have been due partly to adenoids and insufficient oxygenation. There might have been obstruction in the posterior part of the nose, although the result of inspection anteriorly had been negative. The characteristic skin and facial expression of cretinism were absent.

Dr. Sayre suggested enlargement of the air passage by treating the tonsils and adenoids.

Dr. Ketch said it would be of interest to know whether this relief would promote normal growth.

Dr. H. S. Stokes said that the patient was probably a constitutionally lymphatic child, one of a class of patients in whom the administration of anæsthetics was attended with danger. Without hastily making a positive diagnosis of this condition, he suggested that the use of anæsthetics be preceded by a thorough physical examination.

POTT'S DISEASE OR FRACTURE OF VERTEBRAE.

Dr. Townsend presented a girl, 6 years of age, with a very obscure history. Two years ago, when living with her grandmother, after an accident in which she fell down a flight of stairs, striking the back of

---

*Dr. Samuel Ketch died suddenly of cardiac disease on December 14, 1899, at the age of 44. His untimely decease was sincerely mourned by his bereaved colleagues. The meeting of December 15, 1899, was adjourned out of respect to his memory.

her neck, a bony prominence had been noticed, with difficult respiration and a habit of supporting her head with the hand placed under the chin. Kyphosis was marked, as shown by the accompanying cut, involving the sixth and seventh cervical and the first and second dorsal, with a depression of the upper cervical vertebræ.

Dr. A. B. Judson thought that the number of the involved vertebræ pointed away from fracture and towards Pott's disease. The elements of diagnosis in orthopaedic cases might be arranged in the following order of relative importance: First, signs (objective); second, symptoms (subjective); third, history as given by the mother; and fourth, history as given by the grandmother.

Pott's Disease, or Vertebral Fracture.

Dr. Sayre said that a forward position of the head in cervical Pott's disease was frequently attended by difficult breathing. He thought, however, that the child had suffered a fracture and recalled the case of a man who had fallen down stairs striking the back of his head. Partial paralysis of the arms developed from pressure. A diagnosis of Pott's disease had been made, but the signs and history indicated a fracture.

Dr. Townsend said that the treatment, at least, was not doubtful. The affected vertebræ should have complete rest, either by a plaster jacket and head spring, or by a posterior spinal support and chin piece. The latter would be less conspicuous and give better support, with or without the addition of supports going up the back of the head, as might be determined by the progress of treatment, which should be prolonged until the disappearance of all signs of an acute condition. Ultimately the patient would carry the head erect without much deformity, as is the rule in cervical disease thus treated.

THE DURABILITY OF THE PLASTER OF PARIS JACKET.

Dr. Stokes related the history of a case of Pott's disease in a girl who was 4 years of age when first seen in September, 1894. Duration of disease, two months. The tenth dorsal vertebra was affected. The plaster of Paris jacket had been applied anew seven times at intervals of from eight to fifteen months, the average being eleven months. No pain or discomfort had been traced to the apparatus. At the last application, on October 13, 1899, it was found that a small stone had slipped into the jacket and caused an erosion, which had healed in a few days.

Dr. Townsend has seen plaster jackets that had been worn two years.

Dr. L. W. Ely cited a case in which the jacket had been re-applied at intervals of thirteen and eight months without excoriation.

Dr. Sayre referred to the case of a child who had worn a solid jacket for two years.

Dr. H. Gibney cited three cases. 1. A boy seen in 1891. Age, 4 years. Location, middle and lower dorsal region. Emaciation. A large psoas abscess. First jacket was worn two months, the second one year, and the third had been applied two months ago. There had been no increase of deformity, the abscess had been to a large extent resolved, and the general health had improved. 2. Boy. 1895. Six years. Tenth dorsal. First jacket worn three months, and second eleven months, and the third was applied three months ago. The local condition was favorable and the health had improved. In the third case, that of a woman of 27 years, a firmly fitting jacket had been worn for a year without inspection, with freedom from pain and discomfort, and with good effect.

Dr. Sayre cited two cases in which patients had not done well with jackets that were removable, but which progressed favorably towards recovery when the immovable dressing had been applied. In both cases the treatment had been modified in its early stages by the overweening kindness of the grandmothers of the children. He had seen cases in which efforts to replace comfortable jackets by new ones had not been brilliantly successful, it having been a long time before the patient was again made comfortable. For obvious reasons a jacket should not remain in place too long on a child who was growing fast.

Dr. V. P. Gibney said that more important than the question of time was that of applying the jacket so as to give good support and avoid excoriations. A jacket well applied would not disturb the skin, and should be durable. In the case reported by Dr. Stokes the trifling excoriation had soon healed and a cure had been effected by the prolonged splinting of the back.

Dr. Sayre said that excoriations could generally be avoided by a careful application of the jacket.

Dr. Stokes said that the percentage of excoriations was small and in ten cases the trouble had been due to the jacket in four cases; and to foreign bodies, little things such as pennies and button hooks, in six cases. Excoriations caused by a jacket were evidences of a want of skill and experience on the part of the surgeon.

Dr. Sayre said that the skin could be kept clean and healthy by passing a whale-bone inside of the jacket and so pulling up and down a fine handkerchief dampened with alcohol.

Dr. H. Gibney said that a solid jacket should be applied over a long strip of six inches wide linen or gauze, which could be daily wet with alcohol and drawn back and forth.

PLASTER OF PARIS COMPARED WITH STEEL APPARATUS.

Dr. Ketch said that the condition of the skin should be made the subject of stated investigation, not to prevent excoriations, but to ascertain whether we were giving the diseased vertebral column all the mechanical support which the toleration of the skin warranted. The use of a steel apparatus facilitated an occasional and desirable estimate of possible decrease or increase of deformity, which was impossible with the immovable dressing. Changes in the shape of the patient, from growth or otherwise, should meet with corresponding changes in the pressure made by the apparatus.

Dr. Townsend said that the frequent removal of the jacket or brace was one of the worst things that could be done. It was not practiced in the treatment of fractures. In Pott's disease we sought proper anchylosis at the seat of disease. We therefore immobilized the vertebral column. So long as the jacket was clean and the skin healthy we could forego the doubtful advantage to be gained by frequent inspection and rely on the effectiveness of the apparatus.

Dr. Ketch said that the removal of the brace for alterations, when done with ordinary care, could not delay or interfere with consolidation. The more scientific procedure was to use an apparatus which was under intelligent surgical control.

Dr. V. P. Gibney, had failed to see that important benefits could be gained by taking off the apparatus from time to time. If sure of the diagnosis and of a well fitting plaster jacket he was confident of a good result.

Dr. Sayre said that in the cervical region, and anywhere above the tenth dorsal, a jacket should be supplemented by the jury-mast and by a brace to control the shoulders. Traction and control of the movements of the head were very important. He often made use of a metal and leather support to make a base for the jury-mast.

Dr. H. Gibney commended the method of application in which the patient rested on two untempered steel rods, bent to fit the shape, and elevated from the table, partly lying on them supine, and partly held up by two assistants who made gentle traction, the rods being drawn out while the plaster was setting.

## Figures and Tables

**Figure f1:**